# Alveolar bone loss is associated with oral cancer: a case-control study

**DOI:** 10.3389/froh.2025.1569491

**Published:** 2025-05-09

**Authors:** Leah Trumet, Roman Fuchs, Joy Backhaus, Bettina Grötsch, Kerstin Galler, Marco Kesting, Manuel Weber

**Affiliations:** ^1^ Department of Operative Dentistry and Periodontology, Friedrich-Alexander-Universität Erlangen-Nürnberg (FAU), Erlangen, Germany; ^2^ Deutsches Zentrum Immuntherapie (DZI) and Comprehensive Cancer Center Erlangen-EMN (CCC ER-EMN), Friedrich-Alexander-Universität Erlangen-Nürnberg (FAU), Erlangen, Germany; ^3^ Department of Oral and Cranio-Maxillofacial Surgery, Friedrich-Alexander-Universität Erlangen-Nürnberg (FAU), Erlangen, Germany; ^4^ Institute of Medical Teaching and Medical Education Research, University Hospital of Würzburg, Würzburg, Germany; ^5^ Department of Internal Medicine 3-Rheumatology and Immunology, Friedrich-Alexander-Universität Erlangen-Nürnberg (FAU), Erlangen, Germany

**Keywords:** OSCC, periodontitis, orthopantomogram, oral medicine, oral health, bone loss

## Abstract

**Background:**

A link between chronic inflammation and malignant transformation is evident in various cancer types. Periodontitis is the most common chronic inflammatory condition in oral medicine with a proven association with systemic diseases like diabetes. Although there is scant evidence of a potential link between periodontitis and oral cancer there is no proof for a correlation yet. We hypothesize that radiographic bone loss (RABL) as indicator of chronic periodontitis is associated with the occurrence of oral squamous cell carcinomas (OSCC).

**Methods:**

206 orthopantomograms (OPTs) from a cohort of OSCC cases and controls without OSCC, both between the age of 40 and 70, were analyzed in this retrospective study. Radiographic oral health parameters like radiographic alveolar bone loss (RABL), remaining teeth as well as implants were analyzed and compared between the two groups. The analyses of the study were controlled for the impact of confounders such as diabetes, smoking of tobacco and age. Welch-test, Chi-Square-Test and a two-way Analysis of Covariance (ANCOVA) followed by a Bonferroni *post-hoc* test for multiple pairwise comparison were performed.

**Results:**

Several statistically significant differences were identified between the two groups, with a greater than twofold prevalence of nicotine consumption among the OSCC group. Additionally, the OSCC cohort exhibited a mean age approximately 3.5 years higher and a lower number of remaining teeth compared to the control group. After eliminating the effect of these confounders, a significantly greater loss of bone mass was observed in the OSCC cohort in comparison to the control cohort.

**Conclusion:**

In consideration of the confounders, patients with OSCC had more bone loss, compared to controls. These data indicate an association between periodontitis derived chronical inflammation and the malignant transformation of oral epithelium.

## Introduction

Oral squamous cell carcinoma (OSCC), a common form of head and neck malignancy, affected approximately 378,000 individuals worldwide in 2020 (1, 2). OSCC typically arises in the oral mucosa and presents clinically as a red or white lesion showing undefined borders and with an irregular surface (1). The most prevalent site of OSCC is the border of the tongue, followed by the floor of the mouth and other regions, including the soft palate and gingiva (1).

There are several recognized risk factors for OSCC, including smoking and nicotine abuse (1, 3, 4). It is generally accepted that inflammatory and malignant processes are associated with one another (5–7). This hypothesis is corroborated by recent studies which have also suggested periodontal disease (PD) as a co-risk factor for the development and progression of OSCC (2–4, 8). PD is one of the most prevalent chronic inflammatory diseases globally, and it is linked to alterations in the immune system at both the local and systemic levels (4, 7–10). In Germany, more than 50% of young adults (35–44 years of age) and 64% of elderly people (65–74 years of age) suffer from moderate or severe periodontitis (11).

Studies have not only focused on the interplay between inflammation and malignancy, but have also shown that that the duration of inflammation may also increase the risk of developing cancer (4, 6). Given that a chronic inflammatory state persists in PD, several mechanisms may contribute to the development of oral tumors (4, 8). PD may be pro-tumourigenic through increasedactivation of pro-inflammatory signaling pathways as well as the activation of immunosuppressive Checkpoints that promote immune evasion (4, 12). This could lead to inhibited apoptosis, increased proliferation of dysplastic or malignant cells and a reduced efficiency of immunological clearance of malignant cell clones (4). These PD-associated, immune-mediated effects extend beyond the periodontal tissue and can also be detected systemically (13).

PD is induced by a dysbiotic biofilm in the oral cavity and in gingival pockets, which triggers an immune response in adjacent tissues. If the immune response is extensive or inadequate, a chronic inflammatory state develops, leading to the degradation of the periodontal ligament and to resorption of alveolar bone (4, 7, 9, 10, 14, 15). Clinically, this is mainly detected by increased periodontal probing depth and bleeding on probing, in later stages by increased tooth mobility and eventually tooth loss.

As parameters such as bone resorption are observable by radiographic imaging, numerous research groups have analyzed orthopantomograms (OPTs) to examine oral health data (14–17). Currently, the assessment of radiographic alveolar bone loss (RABL) is mainly based on panoramic radiographs, bitewing radiographs and periapical images (15–17).

A review of 13 studies suggests a possible association between PD, as indicated by RABL and bleeding on probing, and the possible presence of OSCC (18). A retrospective study found higher radiologic bone loss in OSCC patients compared to a non-tumor control cohort (5). In their case-control study, Shin et al. suggested a potential association between periodontitis and OSCC (19). They observed that patients with radiologic evidence of pathological alveolar bone loss were almost four times more likely to have OSCC than those without (19). It should be noted that the number of controls examined in this study was approximately twice the number of OSCC patients (19).

Further studies suggest that the causal relationship between the severity of periodontitis and the risk of OSCC is significant, even after adjusting for traditional confounders such as smoking and alcohol consumption (8). In addition, it was observed that tooth loss due to PD is associated with an increased risk of head and neck cancer (8).

Although there are now several studies that have described an association between periodontitis and OSCC, an analysis that accurately quantifies the relationship between alveolar bone loss and the diagnosis of OSCC, taking into account confounding factors such as smoking, diabetes, age and tooth loss, is still lacking.

Therefore, the aim of this retrospective case-control study is to analyze the RABL in OSCC patients and an age-matched control group in relation to the relevant confounders as well as considering parameters of tumor malignancy including tumor size (pT-Status) and lymph node metastases (pN-Status) in terms of a multivariate statistical analysis.

## Materials and methods

### Study design

The Case-Control study was conducted according to the STROBE recommendations (Strengthening the Reporting of Observational Studies in Epidemiology).

### Patients collective

**Figure 1:**
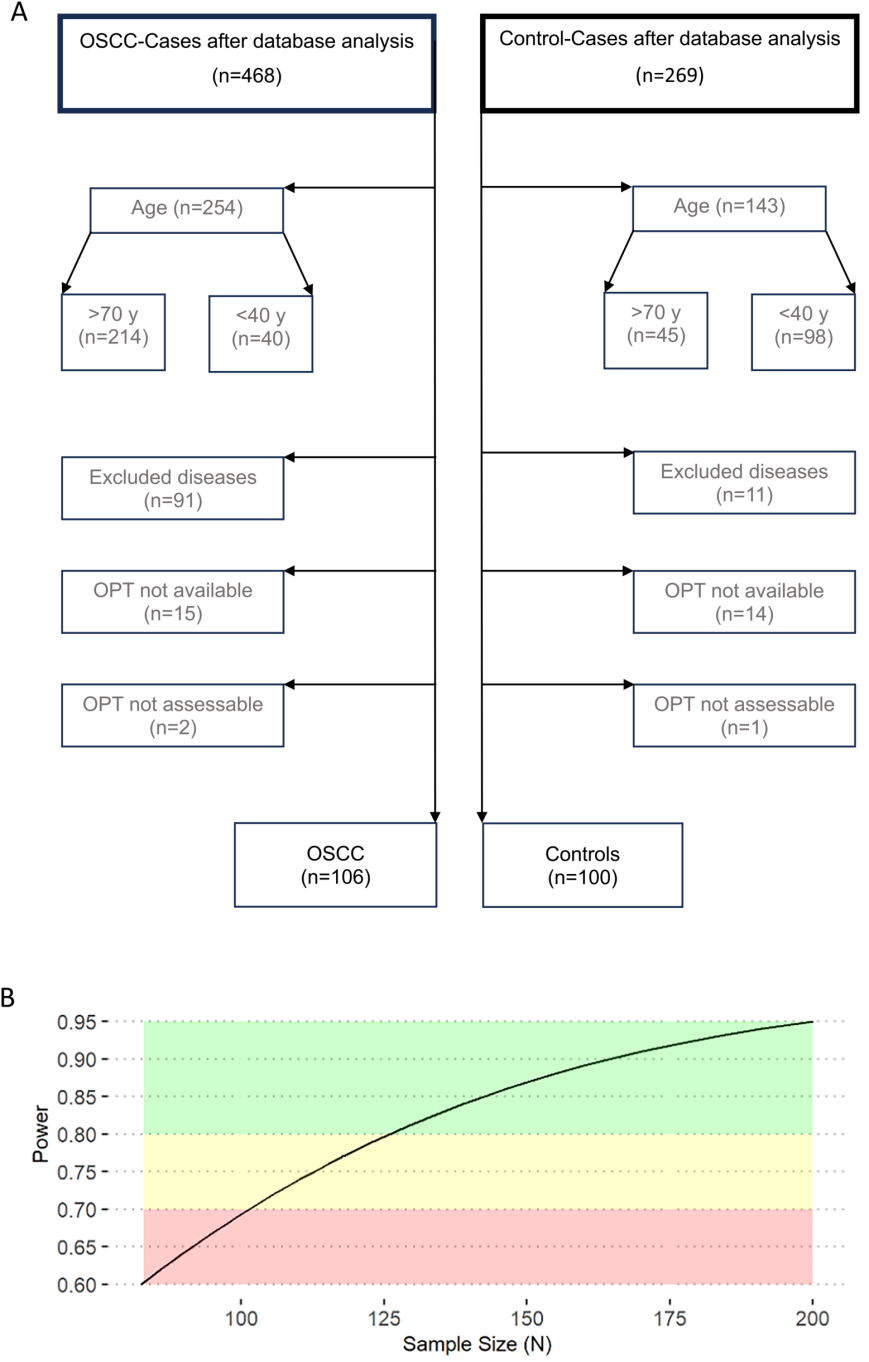
Selection of cases and statistical power calculation. This figure shows the cases selection process **(A)** and the statistical power in relation to sample size (*N*) for a Two-Way ANCOVA analysis **(B)**. For an alpha-probability rate of 0.05, a medium effect size across hypothesis, a power of 0.95 and three covariates a sample size of 200 was considered to be sufficient for valid ANCOVA results. OPT, orthopantomogram.

For this study, Orthopantomograms (OPTs) of 206 patients of the department of Oral and Cranio-Maxillofacial Surgery the University Hospital Erlangen, Germany were retrospectively selected and analyzed. 106 patients had been diagnosed with primary OSCC and 100 individuals with non-malignant and non-inflammatory maxillofacial diseases (e.g., facial fractures). Patients aged between 40 and 70 years at the time of study inclusion (01/2019–12/2023) were consecutively enrolled. The cohort was divided into two groups, healthy and non-healthy with respect to OSCC (controls and OSCC cases). Exclusion criteria in both groups were previous malignant diseases, any history of autoimmune diseases, chronic inflammatory diseases, osteoporosis, history of medication with antiresorptive, antiangiogenetic, immunosuppressive or immunomodulating drugs. The selection process for included patients is shown in Figure 1A.

All patients were classified according to sex, diabetes, nicotine abuse, remaining teeth, implants and root residues. Nicotine abuse was defined as current status of smoking or a history of smoking of more than 2 packyears. Detailed information on the cohort and furthermore on clinical and histomorphological parameters and on the cohort can be found in Table 1.

**Table 1:** Characteristics of variables according to tumor cases and controls.

*M* ± SD/*n* (%)	Overall (*n* = 206)	OSCC (*n* = 106)	Controls (*n* = 100)	*p* value
Age	57.31 ± 7.65	58.98 ± 6.48	55.54 ± 8.39	**0.001**
Sex				0.816
Male	153 (74.3)	78 (73.6)	75 (75.0)	
Female	53 (25.7)	28 (26.4)	25 (25.0)	
Diabetes				0.606
No	183 (88.8)	93 (87.7%)	90 (90.0)	
Yes	23 (11.2)	13 (12.3)	10 (10.0)	
Nicotine abuse				0.001
No	146 (70.9)	62 (58.5)	84 (84.0)	
Yes	60 (29.1)	44 (41.5)	16 (16.0)	
Remaining teeth	20.44 ± 8.81	18.58 ± 9.54	22.41 ± 7.51	**0** **.** **002**
Implants	0.22 ± 0.81	0.12 ± 0.55	0.33 ± 1.01	0.065
Root residues	0.45 ± 1.52	0.57 ± 1.77	0.32 ± 1.19	0.246
Radiographic Alveolar Bone Loss (RABL) in %	24.42 ± 10.61	29.12 ± 10.88	19.67 ± 7.89	**<0** **.** **001**
Age related bone loss ratio (ABL)	0.8 ± 0.36	0.89 ± 0.36	0.71 ± 0.33	**<0** **.** **001**

The table shows the characteristics of the 206 patients in the study cohort. The included cases are given in total numbers (*n*) and percentage (%). *M*, mean; SD, standard deviation; *n*, number; %, percent column wise for numerical, categorical data respectively. Bold denotes statistical significance (*p* < 0.05).

The study was approved by the Ethics Committee of the Friedrich-Alexander-University of Erlangen-Nuremberg on 29 February 2024 (24-43-B) and follows the World Medical Association's Declaration of Helsinki.

### Examination

Based on the data available from the hospital information system (HIS) at the University Hospital Erlangen, the patients’ sex, age at the time OPTs were taken, presence of diabetes and active nicotine abuse were recorded. All OPTs examined for this study were taken on a Sirona Orthophos XG (Sirona Dental Systems GmbH, Bensheim, Germany) with the settings (14.1 s exposure time, 64 kV, 16 mA) and after positioning the patients as recommended by the manufacturer. The radiographs were analyzed by two dentists using Sidexis XG Version 2.63 (2016 Sirona Dental Systems GmbH) evaluation software. The images were displayed on an EIZO RadiForce MX 242W monitor, which is standardized according to DIN ISO 6868-157.

**Figure 2:**
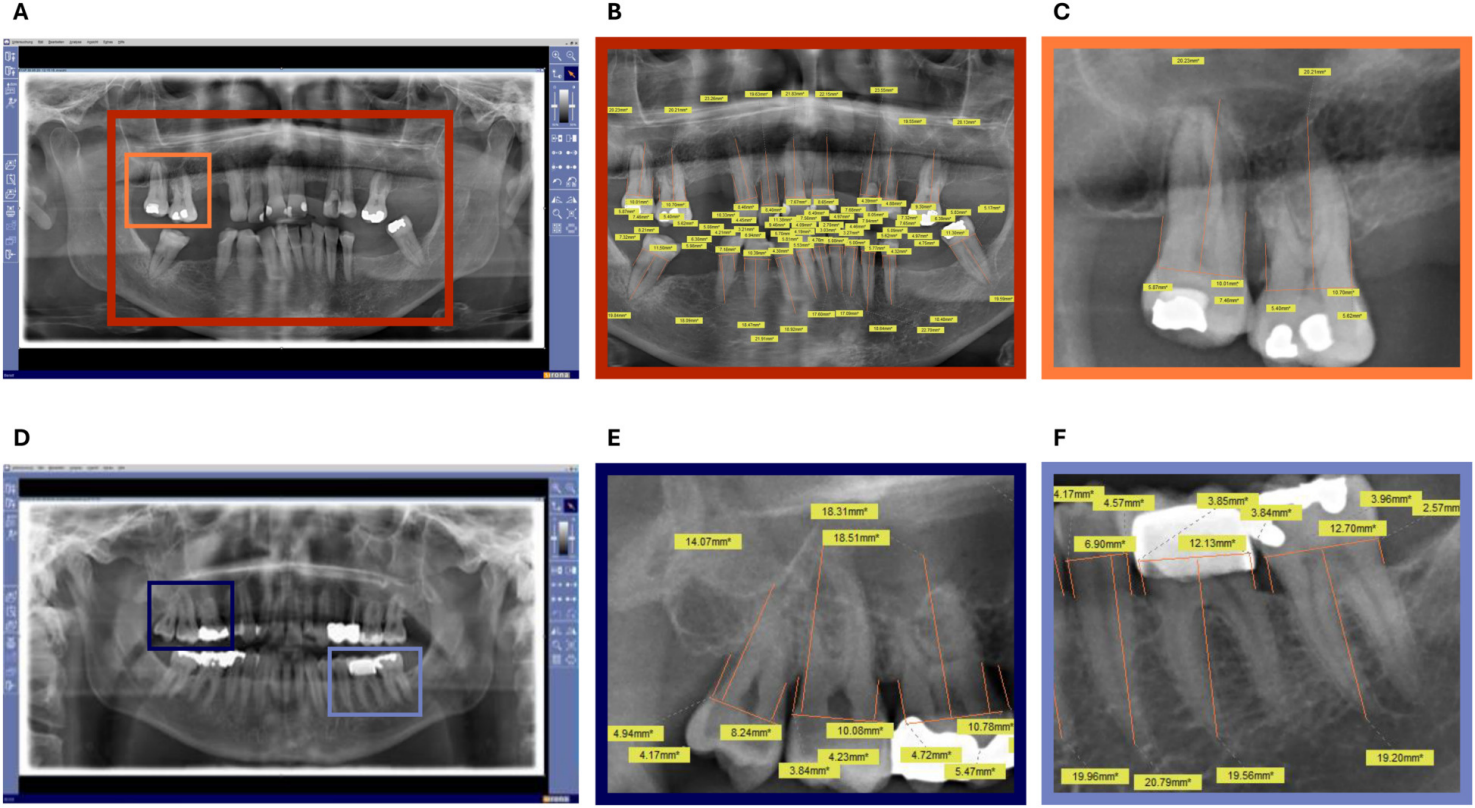
Exemplary OPTs from the analysis for the assessment of radiographic bone loss (RABL). **(A)** Non-edited OPT of a patient in the control group. **(B)** Edited OPT of a patient in the control group. **(C)** Section of the edited OPT of a patient in the control group. **(D)** Non-edited OPT of a patient in the OSCC group. **(E)** Section of an edited OPT of a patient in the OSCC group, showing part of the upper jaw. **(F)** Section of an edited OPT of a patient in the OSCC group, showing part of the lower jaw. OPTs show the unprocessed x-ray images **(A,D)** and a completely measured OPT **(B)** as well as various sections of x-ray images **(C,E,F)** in which the radiographically visible bone resorption was measured. The color-marked frames (light blue, navy blue, yellow and orange) show from which areas of the OPTs the corresponding zoomed-in areas are. The orange lines show the measured distance and the yellow boxes with the number show the corresponding measurement in mm.

In young and periodontally healthy individuals, the alveolar bone level in OPTs is just below the cementoenamel junction. To evaluate radiographic alveolar bone loss (RABL), digital measurement lines were applied to each radiograph using the software's integrated tools. Bone loss was assessed individually for each tooth. First, a horizontal reference line was drawn connecting the cementoenamel junctions on the mesial and distal surfaces of the tooth. Next, a perpendicular line was extended from this reference line to the root apex to establish the total root length. Finally, two vertical lines were drawn parallel to this axis, extending from the cementoenamel junctions mesially and distally toward the crestal bone, in order to quantify bone loss in these respective areas (Figure 2C,E,F). The measured mesial and distal bone loss in mm was then averaged and this value was related to the root length in mm, as surrogate parameter for the bone loss. The calculated % bone loss was termed RABL and was determined for each individual tooth, then averaged for each quadrant and then for the entire dentition. In addition, age-related bone loss (ABL) was determined by dividing the RABL expressed as a percentage of the root length by the age of the patient to provide indirect evidence of PD progression (20) (Table 1).

The analysis of radiographs and data evaluation was based on the 2017 Classification of Periodontal and Peri-Implant Diseases of the European Federation of Periodontology (EFP), where bone loss is considered physiological if overall <15%, moderate if between 15% and 33%, and severe if >33% (20).

### Statistical analysis

#### Hypothesis

Since sample size and statistical analysis irretrievably depends on hypothesis to be falsified, we represent the three main null hypotheses first:

H1_0_: Test and Control Groups do not differ in radiographic alveolar bone loss (RABL) when controlling for the impact of confounders such as diabetes, nicotine abuse and age.
H2_0_: Patients with tumors do not have less teeth compared to their healthy controls when controlling for the impact of confounders such as diabetes, nicotine abuse and age.
H3_0_: RABL is lower for T1/T2 tumors than for T3/T4 tumors when adjusting for the impact of confounders such as diabetes, nicotine abuse and age.
H4_0_: RABL is lower for N0 tumors than for N+ tumors when adjusting for the impact of confounders such as diabetes, nicotine abuse and age.

#### Sample size estimation

For an alpha-probability rate of 0.05, a medium effect size across hypothesis, a power of 0.95 and three covariates a sample size of 200 was computed to be adequate and sufficient (21) to yield valid results within an ANCOVA.

#### Statistics

Welch-test was used to detect differences between two-samples for metric dependent variables. In case of categorical dependent variables Chi-Square-Test was employed. To falsify the null hypothesis 1–4 a Two-way Analysis of Covariance (ANCOVA) was computed. A significant ANCOVA was followed by a Bonferroni *post-hoc* test for multiple pairwise comparisons to pinpoint which groups differ and whether interactions between independent variables appear. Partial Eta Squared (*η*^2^) is reported as an effect size with values of 0.01–0.06, 0.06–0.14 and greater 0.14 considered a small, medium and large effect size respectively (22). To further ease clinical interpretation conditional probabilities are expressed as Adjusted Odds Ratios (aOR).

Outlier were detected using the 1.5 × IQR, 3.0× IQR for normal, extreme outlier respectively (23). Normality is tested using Kolmogorov–Smirnov, nonetheless ANOVA has proven robust against violations of normality (24). Significant values between 0.05 to 0.1, 0.01 to 0.05 and those less 0.01 are referred to as marginally significant, significant and highly significant respectively (25). Sample size calculation is conducted using G*Power (26), statistical analysis are performed with SPSS 29 (IBM), graphical representations are supported by the R-package ggplot2 (27).

## Results

Results consist of two main parts: The descriptive statistics section characterizes participants with a focus on univariate analysis of differences between OSCC and controls. The inferential statistics section tests the four hypothesis and examines interactive effects.

### Descriptive statistics section

#### Patients’ cohort

A sufficient sample size to reach a power of 0.95 at an alpha-error-rate of 0.05 was reached (cf. green area of Figure 1B). This case-control study comprised a total of 206 participants with 106 OSCC cases and 100 matched controls. In the analyzed cohort, 25.7% (*n* = 53) of patients were female and 74.3% (*n* = 153) male (Table 1). The age range was from 40 to 70 years, with a mean age of 57.31 (±7.65) years.

Significant differences between OSCC and control group appeared for the unalterable clinical variable age, which subsequently was treated a confounding covariate in subsequent analysis.

For nicotine abuse a significant difference was determined, with more than twice as many participants abusing nicotine in the OSCC group. Moreover, the Patients of the OSCC group were on average about 3.5 years older and had fewer remaining teeth. Considering the radiographic bone loss of the control group there was one third fewer overall bone loss (cf. Table 1).

#### Distribution of OSCC according to radiographic bone loss (%)

It should be noted in advance that only 100 people in the case group were included in the statistical analysis of bone loss, as six people had fewer than two remaining teeth.

Whereas the majority of OSCC (64%) and controls (63.6%) showed a moderate bone loss (RABL 15%–33%), the relations were reversed for physiological (RABL < 15%) and severe bone loss (RABL > 33%). Physiological bone loss was found in 7% of the OSCC group and 29.3% of controls, whereas severe bone loss was found in the remaining 7.1% of the controls and in 29% of the OSCC (cf. Table 2). A marginally significant association was found between tumor size and RABL with an increasing amount of bone loss in T3/T4 OSCC.

**Table 2:** Tumor characteristics grouped by radiographic bone loss (RABL) (%).

Variable	*n* (%)^d^	Radiographic bone loss (RABL) (%)^d^			
		<15^a^	15.01 ≤ 33^b^	>33^c^	*p* value
		Physiologic	Moderate	Severe	
T-status		7 (7.0)^e^	64 (64.0)	29 (29.0)	0.062
T1/T2	61 (61.0)	6 (85.7)	42 (66.0)	13 (44.8)	
T3/T4	39 (39.0)	1 (14.3)	22 (34.0)	16 (55.2)	
N-status		7 (7.0)	64 (64.0)	29 (29.0)	0.052
N0	64 (64.0)	7 (100.0)	42 (65.6)	15 (51.7)	
N+	36 (36.0)	0 (0.0)	22 (34.4)	14 (48.3)	
L-status		7 (7.1)	62 (63.3)	29 (29.6)	0.679
L0	89 (90.8)	7 (100.0)	56 (90.3)	26 (89.7)	
L+	9 (9.2)	0 (0.0)	6 (9.7)	3 (10.3)	
V-status		7 (7.1)	62 (63.3)	29 (29.6)	0.811
V0	94 (95.9)	7 (100.0)	59 (95.2)	28 (96.6)	
V+	4 (4.1)	0 (0.0)	3 (4.8)	1 (3.4)	
Pn-status		7 (7.1)	62 (63.3)	29 (29.6)	0.093
Pn0	85 (86.7)	7 (100.0)	56 (90.3)	22 (75.9)	
Pn+	13 (13.3)	0 (0.0)	6 (9.7)	7 (24.1)	
Grading		7 (7.1)	63 (63.6)	29 (29.3)	0.045
G1	12 (12.1)	2 (28.6)	9 (14.3)	1 (3.4)	
G2	53 (53.5)	5 (71.4)	35 (55.6)	13 (44.8)	
G3	34 (34.3)	0 (0.0)	19 (30.2)	15 (51.7)	
Recurrence		7 (7.2)	63 (64.9)	27 (27.8)	0.434
No	94 (96.9)	7 (100.0)	60 (95.2)	27 (100.0)	
Yes	3 (3.1)	0 (0.0)	3 (4.8)	0 (0.0)	
Anatomical tumor -localisation		7 (7.0)	64 (64.0)	29 (29.0)	0.230
Palate	4 (4.0)	1 (14.3)	1 (1.6)	2 (6.9)	
Inside of cheek	9 (9.0)	1 (14.3)	7 (10.9)	1 (3.4)	
Tongue	19 (19.0)	4 (57.1)	11 (17.2)	4 (13.8)	
Floor of mouth	24 (24.0)	0 (0.0)	17 (26.6)	7 (24.1)	
Upper jaw	8 (8.0)	0 (0.0)	7 (10.9)	1 (3.4)	
Lower jaw	16 (16.0)	1 (14.3)	9 (14.1)	6 (20.7)	
Tongue and floor of mouth	12 (12.0)	0 (0.0)	7 (10.9)	5 (17.2)	
Inaccurate location	8 (8.0)	0 (0.0)	5 (7.8)	3 (10.3)	
Side of tumor-localisation		7 (7.0)	64 (64.0)	29 (29.0)	0.170
Right	34 (34.0)	1 (14.3)	27 (42.2)	6 (20.7)	
Left	53 (53.0)	5 (71.4)	31 (48.4)	17 (58.6)	
Both sides	13 (13.0)	1 (14.3)	6 (9.4)	6 (20.7)	
Adjuvant therapy		7 (7.0)	64 (64.0)	29 (29.0)	0.004
No adj. therapy	34 (34.0)	6 (85.7)	23 (35.9)	5 (17.2)	
radiotherapy	25 (25.0)	1 (14.3)	18 (28.1)	6 (20.7)	
radiochemotherapy	41 (41.0)	0 (0.0)	23 (35.9)	18 (62.1)	

The table shows the radiographic bone loss (RABL) depending on different analyzed histomorphologic, anatomic, prognostic and therapeutic variables of OSCC. RABL was divided into physiological (a), moderate (b) and severe (c). The total number of cases (*n*) and the percentage (%) in each of the three RABL groups is given. Bold denotes statistical significance (*p* < 0.05).

^a^ RABL < 15%: *n* = 36, 17.4%, considered physiological RABL.

^b^ RABL ≥ 15% and ≤33%: *n* = 127, 61.4%, considered moderate RABL.

^c^ RABL > 33%: *n* = 36, 17.4%, considered severe RABL.

^d^ Column percentage.

^e^ Row percentage.

### Inferential statistics section

In the inferential statistic section main and interaction effects of OSCC and Control Cohort, age and nicotine on RABL, remaining teeth and tumor size were scrutinized.

#### Hypothesis 1: RABL

Statistical analysis indicated that mean overall RABL was not normally distributed (*p* < 0.001). Nicotine abuse was reported by 1 out of 6 individuals in the control group but almost 1 out of 2 in the OSCC group (cf. Table 3). Furthermore, RABL was most prevalent in the OSCC group, where patients also had the lowest numbers of remaining teeth.

**Table 3:** Radiographic bone loss (RABL), age and remaining teeth depending on OSCC diagnosis and nicotine abuse.

Group (*n*)	Nicotine abuse	*M* ± SD		
		Age	RABL (%)	Remaining teeth
Control (16)	Yes	54.1 ± 4.81	18.01 ± 4.81	21.5 ± 6.39
Control (84)	No	55.8 ± 8.24	19.99 ± 8.33	22.6 ± 7.72
OSCC (44)	Yes	57.3 ± 6.30	33.52 ± 12.38	16.4 ± 6.30
OSCC (62)	No	60.1 ± 6.39	26.07 ± 8.56	20.1 ± 8.80

The table shows the age and the radiographic bone loss (RABL) in % and the remaining teeth as mean value and standard deviation depending on the group (controls vs. OSCC) and the state of nicotine abuse.

Significant differences were found between OSCC patients and controls regarding RABL (*η*^2^ = 0.14, *p* < 0.001) with a mean difference of 10%, furthermore age (*η*^2^ = 0.03, *p* < 0.05) and nicotine abuse (*η*^2^ = 0.05, *p* < 0.005), but not for diabetes.

**Figure 3:**
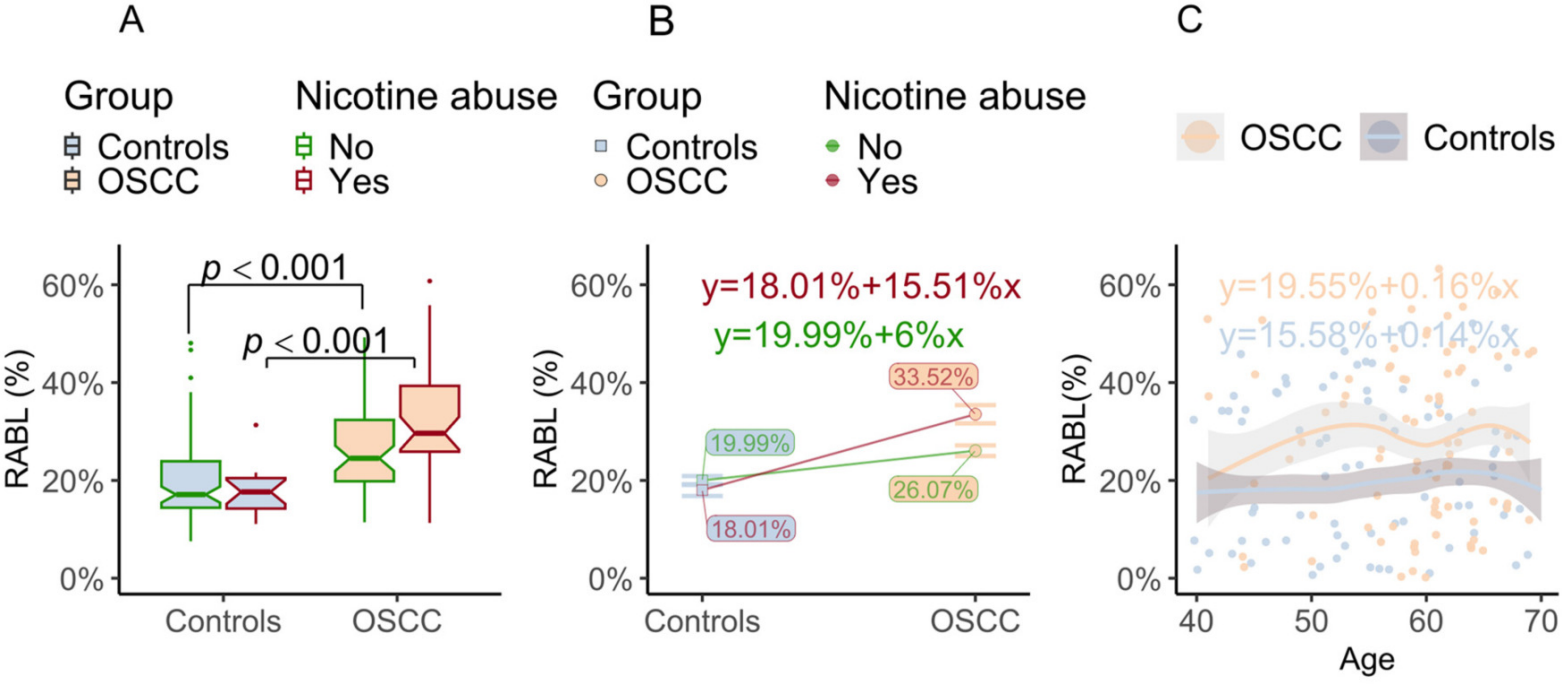
Radiographic bone loss (RABL) depends on OSCC diagnosis, nicotine abuse and age. **(A)** Main significant effect for OSCC when considered in isolation of nicotine abuse: The boxplot shows the radiographic bone loss (RABL) in OSCC cases and controls grouped by nicotine abuse. On the one hand, the figure shows that the RABL is significantly influenced by the group (Controls/OSCC) both in the exclusive comparison of nicotine abuse and in the exclusive comparison of no nicotine abuse. This suggests that the presence of an OSCC significantly influences RABL independently of possible nicotine abuse. *P*-values generated by two-way ANCOVA followed by a Bonferroni *post-hoc* test is indicated. **(B)** Multiplying significant effect of OSCC and nicotine: The figure shows the mean RABL values in the OSCC and Control group in relation to the nicotine abuse status. The straight lines connect the mean values of the same group variable. The difference in the slope, that cause the lines to intersect, indicates an additive effect of smoking on the RABL when comparing the groups. In other words, RABL is exponentially increased by the presence of the tumor and nicotine abuse. The slope (Δ) shown in the equation reflects the effect of the presence of OSCC, which increases by Δ = 15.5% for OSCC compared to controls when abusing nicotine, but only by Δ = 6.0% when nicotine is not abused. **(C)** The slight increase in the straight line shows that there is a small effect of age for RABL in both OSCC and control patients. When comparing the groups, the almost parallel straight lines and their distance from each other show that there is a main effect for belonging to OSCC or Controls group but there is no interactive effect for age on RABL.

There was also a significant interaction between nicotine abuse and the presence of OSCC (*η*^2^ = 0.05, *p* < 0.002), where nicotine abuse multiplied the increase of RABL by the OSCC group by a factor of 2.6 (cf. Figure 3B). As indicated by the effect size, the most important influencing factorfor RABL was presence of OSCC followed by nicotine abuse and age. The null hypothesis is therefore rejected, the alternative hypothesis (H1) is accepted.

Adjusted Odds Ratios for nicotine abuse and severe bone loss indicate that for OSCC patients who abuse nicotine, the risk of severe bone loss is doubled (aOR = 2.77, 95% CI = 1.14–6.74) compared to OSCC patients who did not abuse nicotine. As indicated by the overlapping error bars (c. Figure 3B, left side) the intercepts (*f*) did not differ significantly for controls who abuse nicotine [*f* (0) = 18.01%] and those who did not [*f* (0) = 19.99%], while the difference for nicotine use within the OSCC group with respect to RABL was 7.5%. There was a small effect of age on RABL (see Figure 3C), graphically illustrated by the slope of the parallel lines with gray shaded 95% CIs for OSCC and controls partially overlapping. Age significantly correlated with RABL (*r* = 0.20, *p* < 0.005) but did not enfold an interaction effect with the presence of OSCC.

#### Hypothesis 2: remaining teeth

For the remaining teeth Kolmogorov–Smirnov indicated a violation of normality (*p* < 0.001). Descriptive statistics had already indicated that patients in the OSCC group had less teeth (cf. Table 1). When adjusting for confounding variables, no significant differences were determined between the OSCC and control groups for the number of remaining teeth (*η*^2^ = 0.01, *p* = 0.208).

**Figure 4:**
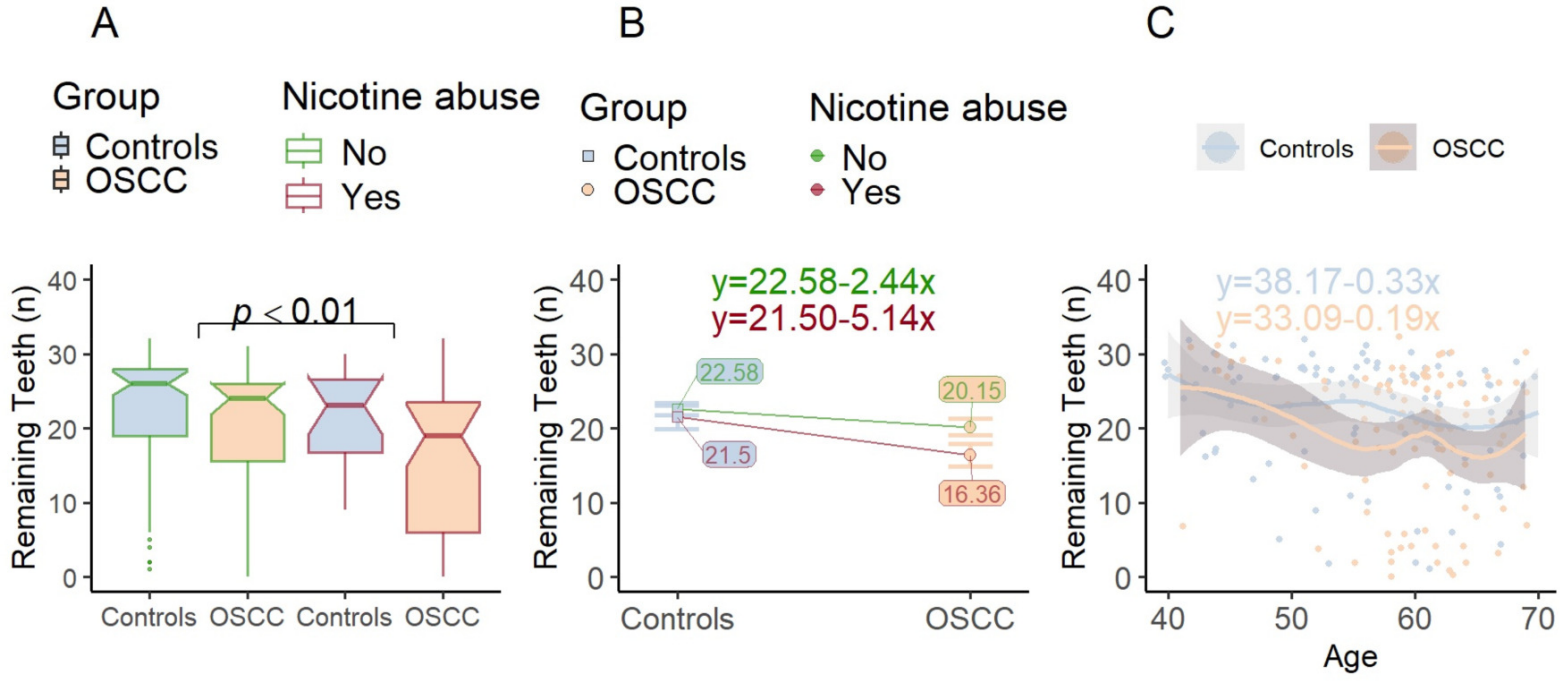
Remaining teeth as a function of nicotine abuse and age. **(A)** In comparison to Figure 3, the mean value bars are arranged so that patients with the same nicotine abuse status are shown next to each other. When patients with and without nicotine consumption are compared with regard to RABL, a significant main effect is shown independent of whether a tumor is present. **(B)** Significant effect of nicotine abuse on Remaining Teeth: The profile plot shows the mean remaining teeth values in the OSCC and Control group in relation to the nicotine abuse status. Remaining Teeth is significantly influenced by nicotine abuse, whereby the effect is more clearly visible within the OSCC group. However, the approximately parallel straight lines show that there is no interaction effect. **(C)** When comparing the groups (Controls/OSCC) the gradient of the parallel lines demonstrates, that there is an effect of Age for Remaining Teeth. It can also be seen that Remaining Teeth is influenced similarly by increasing Age in both OSCC and Control patients. No height difference (in contrast to Figure 3C) can be seen, which indicates that there is no effect for OSCC on the remaining teeth.

It was age (*η*^2^ = 0.06, *p* < 0.001) and nicotine abuse (*η*^2^ = 0.04, *p* < 0.01) which enfold a significant small to medium effect on remaining teeth. This is equally true for the controls as well as the OSCC group (cf. Figure 4A,C). The effect on remaining teeth was significantly more pronounced in the OSCC group (*p* < 0.05), where nicotine users had 3.79 teeth less than individuals without nicotine abuse (Figure 4B). However, considering age, the overlapping gray shaded confidence intervals and the small difference in slope show that there was no significant interaction with the diagnosis of OSCC (Figure 4C).

Hypothesis 2_0_ is verified, patients with tumors do not differ from healthy controls when controlling for nicotine and age regarding the number of remaining teeth.

#### Hypothesis 3: RABL by T1/T2 and T3/T4

**Figure 5:**
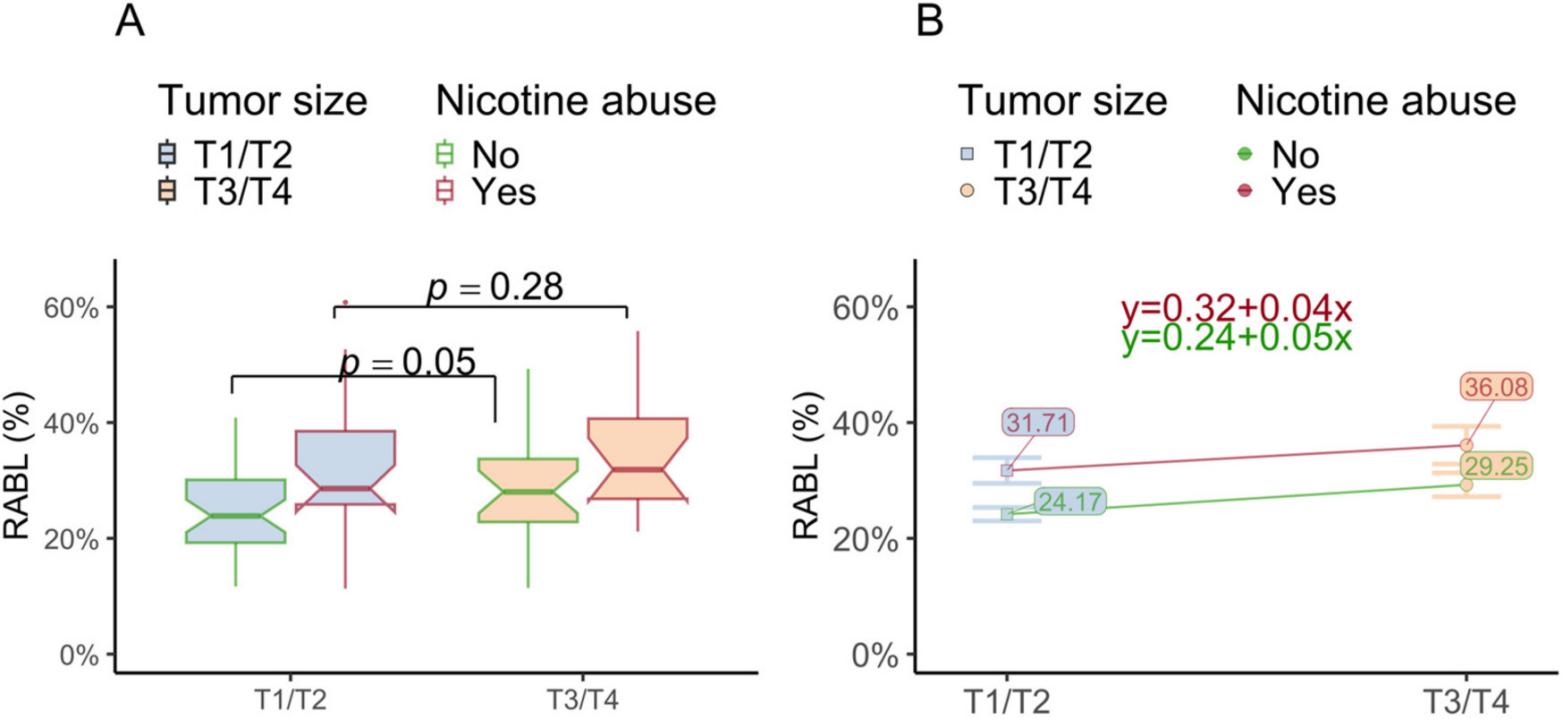
RABL is affected by tumor size and nicotine abuse. **(A)** The box plot shows the radiographic bone loss (RABL) depending on the grouped tumor size (pT1/pT2 vs. pT3/pT4) in relation to the nicotine abuse state. The significant effect for OSCC size on RABL disappears if OSCC patients abuse nicotine. **(B)** The figure shows the mean RABL in small size (pT1/pT2) and large (pT3/pT4) OSCC depending on nicotine abuse. The height difference between the lines for tumor size indicate the strong main effect for nicotine. When comparing only the individuals without nicotine abuse between the different tumor sizes, the tumor size had a significant influence on bone loss. This effect becomes invalid in the presence of nicotine abuse. The equations describe the slope and intercept of the two lines. The lines seem to be nearly parallel indicative for the absence of an interaction effect.

As reported, the first hypothesis Kolmogorov–Smirnov test indicated that RABL was not normally distributed. Patients with larger tumors T3/T4 showed significantly more RABL than patients with T1/T2 tumors when not incorporating age and nicotine abuse (*η*^2^ = 0.05, *p* < 0.05). Whereas a T1/T2 tumor was associated with a RABL of 27.1% (±9.86%), a T3/T4 tumor was associated with a RABL of 32.2% (±11.78%) on average. Although there was a descriptive tendency for the T3/T4 OSCC group abusing nicotine to have greater RABL (cf. Table 4) there was no statistically detectable interaction effect with nicotine. The strong main effect of nicotine suppressed the impact of tumor size. When patients abused nicotine, the effect of tumor size on RABL became non-significant (*p* = 0.28, cf. Figure 5A). Nicotine abuse did not interact with size of tumors (*η*^2^ = 0.00, *p* = 0.87), but showed a strong main effect (*η*^2^ = 0.13, *p* < 0.001). These data show that the effect of nicotine on RABL remained approximately identical regardless of tumor size.

**Table 4:** Radiographic bone loss (RABL) depending on nicotine abuse and tumor size (T1/T2 vs. T3/T4).

pT (*n*)	Nicotine abuse	*M* ± SD
		RABL
T1/T2 (40)	No	24.2 ± 7.37
T1/T2 (27)	Yes	31.7 ± 11.5
T3/T4 (22)	No	29.3 ± 9.62
T3/T4 (17)	Yes	36.1 ± 13.4

The table shows the nicotine abuse state and radiographic bone loss (RABL) in % as mean value and standard deviation depending on the tumor size of OSCC patients represented as pT-Status and grouped in small (pT1, pT2) and large tumors (pT3, pT4).

The latter can be visually examined by the non-intersecting lines in the corresponding profile plot (cf. Figure 5B).

#### Hypothesis 4: RABL by N0/N±

**Figure 6:**
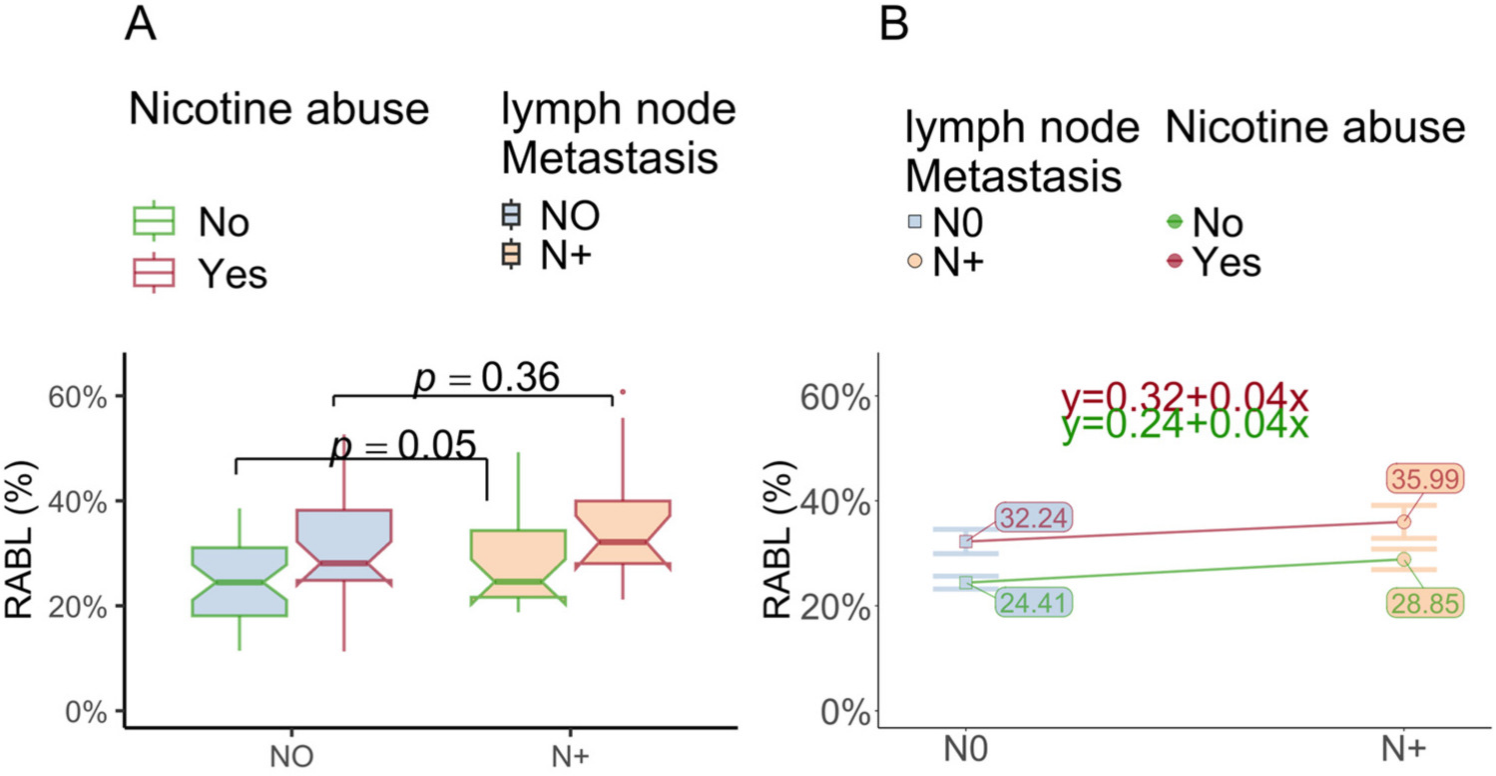
RABL is affected by lymph node metastasis (N+) and nicotine abuse. **(A)** The box plot shows the radiographic bone loss (RABL) depending on the histologic lymph node status (pN0 vs. pN+) in relation to the nicotine abuse state. The significant effect for OSCC N-Status on RABL disappears if OSCC patients abuse nicotine. **(B)** The figure shows that the RABL grouped by the histologic lymph node status (pN0 vs. pN+) of OSCC depending on nicotine abuse. The height difference of the parallel lines indicates the strong main effect for nicotine. When comparing only individuals without nicotine abuse between the state of lymph node metastasis, N-status had a significant influence on bone loss. Like Figure 5, the lines seem to be nearly parallel, which visualizes the absence of an interaction effect.

A marginally significant main effect on RABL for N-status (*η*^2^ = 0.03, *p* = 0.05) was determined as well. Analog to tumor size (c. hypothesis 3), RABL was most pronounced in N+ patients who did abuse nicotine (cf. Table 5) and least in N0 patients who did not abuse nicotine. In line with hypothesis 3 there was a strong main effect of nicotine abuse (*η*^2^ = 0.12, *p* = 0.001). Once nicotine abuse was added to the equation, the difference between N0 and N+ disappeared (cf. to Figure 6A). The latter is graphically reflected by the parallel lines in the profile plot (cf. Figure 6B).

**Table 5:** Radiographic bone loss (RABL) depending on the pN-Status (N0 vs. N+).

pN (*n*)	Nicotine abuse	*M* ± SD
		RABL
N0 (39)	No	24.4 ± 7.69
N0 (29)	Yes	32.2 ± 12.5
N+ (23)	No	28.9 ± 9.39
N+ (15)	Yes	36.0 ± 12.1

The table shows the nicotine abuse state and radiographic bone loss (RABL) in % as mean value and standard deviation depending on the nodal status of OSCC patients represented as pN-Status and grouped non metastasized cases (pN0) and cases with cervical lymph node metastases (pN+).

## Discussion

The current study demonstrated a significant association between increased radiographic alveolar bone loss (RABL) and the diagnosis of oral cancer (OSCC). Increased RABL is one of the main indicators for periodontitis (PD) (28). The main advantages of the ANCOVA analysis used are that interacting effects of covariates can be included in the statistics, effects of individual variables can be considered in isolation from confounders and their interpretation is facilitated by visualizations. These confounding factors such as smoking, diabetes and age increase the risk of both malignant diseases and PD (29). Therefore, these factors must be taken into account when investigating the bidirectional relationship between these two diseases (29). The association between PD indicated by RABL and OSCC, detected in the current study was independent from confounders such as age, diabetes or nicotine abuse. This finding is relevant because both of these cofactors alone have been shown to be associated with the development of both periodontitis and OSCC (30). The use of two-way Analysis of Covariance (ANCOVA) to falsify null hypothesis 1–4 in our study disentangled the differential effects of the association between PD, OSCC and the cofactors. Furthermore, this serves the purpose not only to inspect predictor and criterion relationships but also the associations among these and thereby conditional probabilities. High RABL was significantly associated with OSCC. Smoking showed an independent additive effect with the highest RABL detectable in the group of OSCC patients with nicotine abuse. When the diagnosis of OSCC and smoking were combined, there was an interaction effect, meaning that the effect of nicotine abuse on RABL was not only additive but exponential. Increased age was associated with increased RABL, but there was no significant difference in the influence of this cofounder between the OSCC and the control group. Moreover, the fact that belonging to the group of OSCC or controls has the strongest effect and was independent of the co-founders’ such as age and nicotine abuse indicates that PD associated RABL could be directly linked to an increased risk of developing OSCC. This finding could be explained by chronic inflammatory processes prevalent in periodontitis affected gingiva and oral mucosa.

There is evidence in the literature for several mechanistic links between PD and OSCC development. Serum levels of IL-17A, TGF-β1, were was shown to be significantly increased in OSCC patients (31); this shift in serum immune response that can also be found to some extend in PD patients (32–34). It is generally accepted that local IL-17 signaling is increased in PD affected mucosa (33, 35–37). There is evidence that high IL-17 expression in OSCC tissue is associated with lymph node metastases and inferior survival in OSCC (38). IL-17 could promote tumor initiation and tumor progression by inducing pro-inflammatory and growth-promoting cytokines and mobilizing myeloid cells with suppressive activities to the tumor site (39).

Oral cancer precursor lesions that subsequently underwent malignant transformation had a significantly increased infiltration of inflammatory cells such as macrophages, along with increased expression of the immunosuppressive immune checkpoint ligand PD-L1 and its receptor PD1 (40, 41). This indicates that changes in the local immune environment precede oral epithelium malignant transformation and oral cancer initiation (40, 41). In a xenotransplant mouse model, experimental PD was able to promote tumor growth. Immunofluorescence showed an increased infiltration of PD-L1^+^ tumor associated macrophages and PD-1^+^CD8^+^ T-cells, but a decline of IFN-γ^+^CD8^+^ T-cells in both xenografts and clinical OSCC samples with PD (12). The tumor-promoting and immune-evasive effect of PD was reduced in response to anti-PD1 therapy in the animal experiment (12). PD-related changes in the oral microbiota may contribute to the association between PD and OSCC. For example, the PD-associated bacterium Porphyromonas gingivalis (P. gingivalis) is thought to be a major factor in the upregulation of the PD1/PD-L1 Immune Checkpoint axis (42). Elevated levels of periodontal pathogens such as P. gingivalis and F. nucleatum were identified in tissue samples obtained from patients diagnosed with different types of cancer (8). A higher expression of P. gingivalis and Candida albicans was also observed in patients with OSCC compared to patients without OSCC, indicating a potential association between the two diseases via PD derived alterations of the oral microbiota (1, 7, 8). Previous studies have suggested a possible association between PD and an increased risk of developing oral cancer, with the risk appearing to be approximately three times higher in patients with PD (4).

Besides a relationship between PD and the local risk for cancer development, there might also be systemic associations between periodontitis and malignancies. A current meta-analysis showed an association between PD and the development of lung cancer (29). PD has also been discussed in the context of its potential role as a risk factor for the development and worsening of the prognosis of various cancer other types, like tumors of the breast, pancreas and colon by creating an inflammatory milieu (3, 4, 7, 8, 29).

Considering the interaction effect between OSCC and nicotine abuse, which indicates that patients who have a tumor and consume nicotine have a particularly strong PD indicated by RABL, it could be suggested that the two diseases combined maximize the influence of the risk factors. One possible mechanism for this at the histologic level would be that the weakened oral mucosal barrier caused by PD facilitates the penetration of carcinogenic toxins and pathogens (43). However, it should be noted that this analysis could not demonstrate that the risk of OSCC is increased by the presence of a stronger RABL, but only that there is a strong association.

Analysis of remaining teeth showed that OSCC patients did not differ from healthy controls when controlling for nicotine and age. For the number of remaining teeth, the effect of belonging to the OSCC group could be excluded completely. This study shows that not the affiliation with test or control group accounts for differences int terms of remaining teeth, but solely the confounding variables. If we had only referred to the adjusted Odds Ratio, we would not have been able to isolate the effects of the risk factors. An example of this would be that the influence of nicotine abuse could also have been based on a generally increased age of smokers which could be ruled out by our study. We hypothesize that any study showing a relation between remaining teeth and OSCC solely relying on Odds Ratio could be a result of an insufficient statistical approach.

Studies indicate that good general oral hygiene, characterized by regular tooth brushing, flossing, and dental check-ups, is associated with a higher number of remaining teeth. Poor oral hygiene, on the other hand, is linked to fewer remaining teeth and increased risk of dental issues such as caries and PD (44, 45). Poor oral hygiene might also be associated with an increased risk for oral cancer (46, 47). As the remaining teeth did not differ between OSCC and controls it is unlikely that a general state of poorer oral health is prevalent in the OSCC group accounting for OSCC development and the detected higher level of RABL. Rather, these results support the hypothesis that the presence of PD is associated with the development of oral cancer. In contrast to OSCC diagnosis it was age and nicotine abuse which showed a significant small to medium effect on remaining teeth. This is equally true for the controls as well as for OSCC cases.

**Figure 7:**
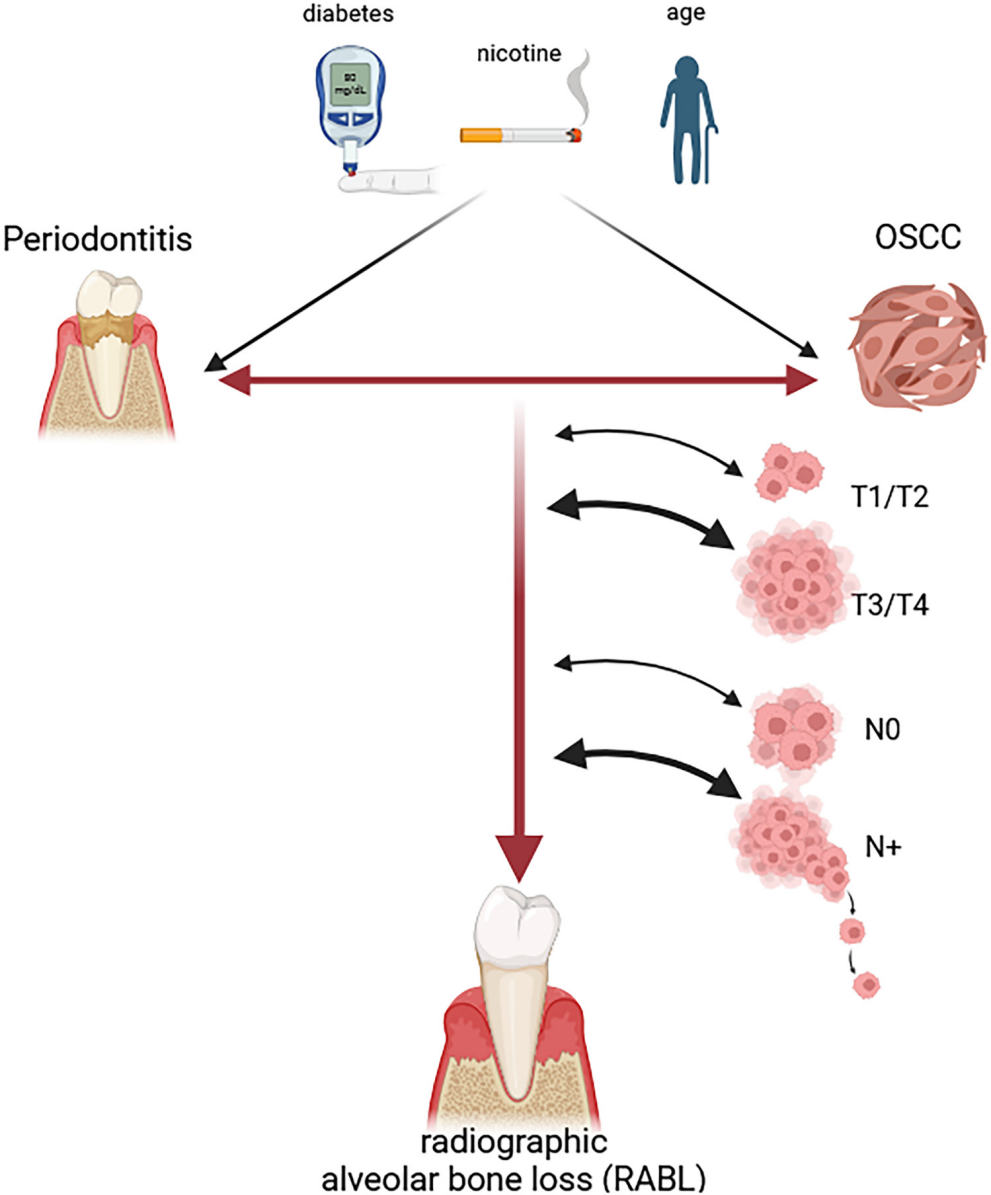
Oral cancer is associated with increased radiographic alveolar bone loss (RABL). The figure shows schematically the results of the current study. Radiographic alveolar bone loss was significantly higher in OSCC patients compared to controls statistically independent of their nicotine abuse status. This indicates a relationship between periodontitis which is the cause for RABL and OSCC. Nicotine abuse was shown to have an additive effect to RABL with the group of smoking OSCC patients showing the highest degree of bone loss. RABL was also significantly increased in larger tumors (pT3/pT4 OSCC) and metastatic cases (pN+ OSCC) in cases with no nicotine abuse. Higher age was shown to increase RABL independent of the belonging to the OSCC or control group. In contrast, diabetes showed no significant influence on RABL. In conclusion, the chronic inflammation caused by periodontitis might facilitate OSCC development and could promote parameters of malignancy including higher pT and pN-Stage.

The results of the current study also indicate, that RABL is associated with higher tumor staging expressed by the association between high RABL and larger pT3/pT4 tumors as well as metastasized pN+ malignancies in the group on non-smoking OSCC patients. The association between tumor size and RABL could also be interpreted as a group of patients showing up with larger tumors might be less aware of oral health and is less likely to consult the dentist with oral symptoms which might be associated with increased PD progression as well as a delayed diagnosis of OSCC. However, the fact that the number of remaining teeth did not differ between OSCC and controls contradicts this interpretation. The increased RABL in pN+ cases in non-smoking OSCC patients might indicate that increased malignancy of the tumors might be associated with the presence of PD. The findings and interpretations of the current study are summarized in Figure 7.

In the current study, OPTs were used for a retrospective evaluation of RABL. Conventional digital intraoral radiographs are considered to be the best choice for radiologic assessment of PD because they provide a high image resolution with minimal radiation exposure (15). OPTs are considered to be slightly inferior but provide a comprehensive assessment of the teeth, alveolar bone and the surrounding structures in a single tomographic radiograph (15). Despite this potential limitation, OPTs were used in the current study as they were available for all OSCC patients and controls, whereas intraoral radiographs were only available in a small proportion of patients. CT scans were not used for RABL detection as they were not available for all controls and high contrast artifacts were present in a proportion of patients with metallic dental restorations.

### Limitations of the study

This study has a few limitations. The data were collected retrospectively, so no clinical assessment of PD was possible and the RABL was used as surrogate marker for presence and severity of PD. In addition, clinical follow-up of bone resorption could not be studied because the data collection was done at one point in time. However, as bone loss occurs over a longer period of time, the one-time point in time is sufficient for this data collection.

## Conclusion

The current study indicates that periodontitis, as measured by radiographic alveolar bone loss, is associated with the development of oral cancer independent of confounding factors such as smoking, age, and diabetes. This may be explained by the pro-tumorigenic effects of chronic periodontal inflammation. Future studies should verify these results in a prospective design and also try to analyze the underlying cellular and immunological mechanisms. As periodontitis is a disease that goes far beyond tooth loss, clinicians should be aware of the potential severity of the disease and try to optimize periodontal treatment.

## Data Availability

The raw data supporting the conclusions of this article will be made available by the authors, without undue reservation.
